# Structural and antigenic variations in the spike protein of emerging SARS-CoV-2 variants

**DOI:** 10.1371/journal.ppat.1010260

**Published:** 2022-02-17

**Authors:** Anshumali Mittal, Arun Khattri, Vikash Verma

**Affiliations:** 1 Department of Structural Biology, University of Pittsburgh School of Medicine, Pittsburgh, Pennsylvania, United States of America; 2 Department of Pharmaceutical Engineering and Technology, Indian Institute of Technology (BHU), Varanasi, India; 3 Biology Department, University of Massachusetts, Amherst, Massachusetts, United States of America; NYU Langone Health, UNITED STATES

## Abstract

The Severe Acute Respiratory Syndrome Coronavirus 2 (SARS-CoV-2) virus is continuously evolving, and this poses a major threat to antibody therapies and currently authorized Coronavirus Disease 2019 (COVID-19) vaccines. It is therefore of utmost importance to investigate and predict the putative mutations on the spike protein that confer immune evasion. Antibodies are key components of the human immune system’s response to SARS-CoV-2, and the spike protein is a prime target of neutralizing antibodies (nAbs) as it plays critical roles in host cell recognition, fusion, and virus entry. The potency of therapeutic antibodies and vaccines partly depends on how readily the virus can escape neutralization. Recent structural and functional studies have mapped the epitope landscape of nAbs on the spike protein, which illustrates the footprints of several nAbs and the site of escape mutations. In this review, we discuss (1) the emerging SARS-CoV-2 variants; (2) the structural basis for antibody-mediated neutralization of SARS-CoV-2 and nAb classification; and (3) identification of the RBD escape mutations for several antibodies that resist antibody binding and neutralization. These escape maps are a valuable tool to predict SARS-CoV-2 fitness, and in conjunction with the structures of the spike-nAb complex, they can be utilized to facilitate the rational design of escape-resistant antibody therapeutics and vaccines.

## 1. Introduction

Enveloped viruses are found across diverse viral families and are characterized by having a lipid bilayer (envelope) derived from the host cell membrane, which contains virus-encoded membrane protein (M), envelope protein (E), and glycoprotein peplomer or commonly known as spike protein (S). These peplomers are often seen as projections from the outer surface of the envelope and are essential for host cell recognition, fusion, and virus entry [1]. The spike glycoprotein is the dominant exposed antigen on enveloped viruses, which can trigger a series of adaptive immune responses mediated by 3 major cell types: B cells (humoral immunity) and CD4^+^ and CD8^+^ T cells (cell-mediated immunity) [1,2]. B cells activation in response to viral infection results in the production of antigen-specific antibodies that can neutralize and clear the virus particle. Viruses also develop immune evasion strategies for escaping these responses through several ways, such as antigenic shielding by the addition of complex glycans [3,4], by secreting truncated viral glycoproteins that share viral spike epitopes for subverting the host immune response [5], by antigenic variations [6], and by blocking complement activation and neutralization of virus particles [6]. On the other hand, activated B cells within germinal centers of secondary lymphoid organs produce class-switched memory B cells, which undergo rounds of population expansion, somatic hypermutation, and selection for improved antigen binding. These evolutionary strategies are regularly used by our immune system to protect us against virus variants and other pathogens [7–9]. In the ongoing Coronavirus Disease 2019 (COVID-19) pandemic, we are witnessing all these features of the human immune system along with continuously emerging Severe Acute Respiratory Syndrome Coronavirus 2 (SARS-CoV-2) variants.

**Fig 1:**
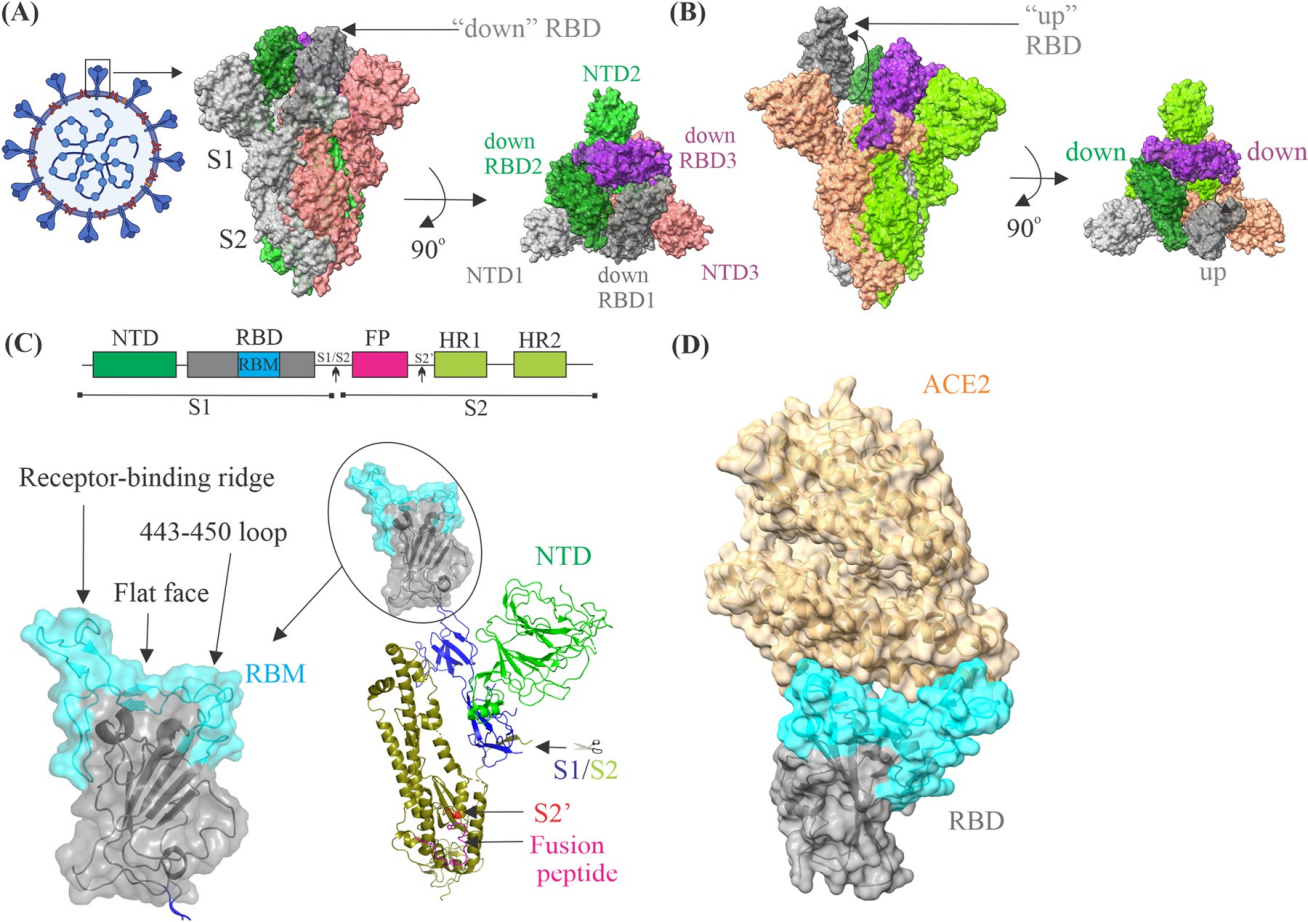
Structure of the SARS-CoV-2 spike protein trimer. (A) Left: side view of the trimeric spike ectodomain with 3 RBDs in the down-conformation; right: top view of the trimeric spike protein showing RBDs in gray, forest green, and orchid (PDB: 6VXX). (B) Left: side view of the trimeric spike ectodomain with 1 RBD in the up-conformation; right: top view of the trimeric spike protein showing 1 up RBD in gray (PDB: 7BNN). (C) A schematic layout of the spike protein is shown at the top. Right: structure of a monomer displaying the RBD in the open conformation. Spike protein structure shows the receptor-binding subunit S1 and the membrane-fusion subunit S2 separated by the furin-like protease site (S1/S2). Different subdomains of the spike protein are the NTD in green, RBD in gray containing RBM in cyan at their top, the fusion peptide in pink, second cleavage site S2’ in red, and HR1 and HR2 in olive (PBB: 7BNN). The scissors represent the S1/S2 boundary at amino acid position 685. Left: The open conformation RBD highlights the 3 different regions: receptor-binding ridge, flat surface, and 443–450 loop of the RBM that form the ACE2-binding region. ACE2, angiotensin-converting enzyme 2; FP, fusion peptide; NTD, N-terminal domain; RBD, receptor-binding domain; RBM, receptor-binding motif; SARS-CoV-2, Severe Acute Respiratory Syndrome Coronavirus 2.

The COVID-19 pandemic caused by the SARS-CoV-2 virus has claimed over 5 million lives worldwide since the first reported case in Wuhan, China [10,11]. Each SARS-CoV-2 contains 24 to 40 randomly arranged spike-like projections on its surface [12,13], which resemble a solar corona under the electron microscope, from which they get their name and are referred to as coronaviruses. The SARS-CoV-2 spike glycoprotein is a homotrimer composed of S1 and S2 subunits separated by a furin cleavage site (RRAR) that modulates its fusogenic activity [14]. The receptor-binding domain (RBD) and N-terminal domain (NTD) of the S1 subunit guide the SARS-COV-2 attachment to host cells, and the S2 subunit drives fusion between virus and host cell membrane [15–18]. Cryo-EM studies have indicated that the SARS-CoV-2 spike protein is wildly flexible and it exhibits several prefusion conformations where 3 RBDs adopt distinct orientations: “up” (receptor-accessible state) and “down” (receptor-inaccessible). Protomers with the up-conformation can facilitate the binding between the spike protein and the angiotensin-converting enzyme 2 (ACE2) receptor (Fig 1). The RBD and ACE2 binding interface shows highly complementary electrostatic surface potentials with the RBD and ACE2 being positively and negatively charged, respectively [13,17,19–21]. The antigenic nature together with multiple conformations of the trimeric spike protein make it an attractive target for drug and vaccine development projects.

RNA viruses, usually approximately 10,000 nucleotides long, exploit various tools of genetic variation for ensuring their survival, which are characterized by high mutation rates, high yields, and short replication times [22]. The low replicative fidelity armors RNA viruses to adapt to different replicative environments and selective pressures, which, in turn, enable them to escape host immunity and develop drug resistance. SARS-CoV-2 encodes an RNA proofreading system (nsp14-ExoN), but despite this, more than 25,000 mutations have been reported in the SARS-CoV-2 genome (https://ngdc.cncb.ac.cn/ncov/variation/annotation). The first notable mutation discovered was D614G in early 2020 [23], and since then, several SARS-CoV-2 variants have emerged periodically, including the Delta (B.1.612.2) [24] and Omicron (B.1.1.529) [25] variants. It is critical to identify these mutations on the RBD that escape antibody-mediated SARS-CoV-2 neutralization. Mutational antigenic profiling has successfully been used for identifying such hotspots in envelope or spike proteins that escape antibody binding in several viruses, including SARS-CoV-2 [26–36]. These antibody escape maps define functional binding epitopes and predict which mutations are selected when the virus is exposed to convalescent plasma or monoclonal antibodies (mAbs). These escape maps in combination with atomic structures could be of great importance from a therapeutic standpoint. Structural studies of the SARS-COV-2 variants spike protein have revealed that they exhibit higher conformational heterogeneity than observed for the Wuhan-Hu-1 isolate or D614G, which might play key roles in binding and locating the ACE2 receptors on cell surfaces [21]. These variants appear to have increased transmissibility, potentially higher pathogenicity and show reduced sensitivity to neutralization by therapeutic mAbs and serum-derived polyclonal antibodies [37–41]. In this review, we discuss the molecular characteristics of naturally occurring SARS-CoV-2 variants including the Delta and Omicron variants that armor them for escaping the therapeutic antibodies, natural infections, or even current vaccines. We define the structural analysis and classification of the RBD targeting antibodies to understand the molecular mechanisms of neutralization and potency differences among them. We further describe the identification of the RBD escape mutations for several antibodies that resist vaccine-elicited and therapeutically relevant antibodies binding with a focus on the SARS-CoV-2 variants.

## 2. SARS-CoV-2 variant classifications and definitions

All viruses acquire mutations as they evolve, and most mutations have either deleterious or neutral effects on viral fitness. However, certain amino acid substitutions, insertions, and deletions give the viruses a selective advantage, which potentially makes them more infectious, transmittable, and, ultimately, the dominant strain. This indicates that negative selection reduces genetic diversity and shapes the evolution of the virus, while positive selection affects only a small number of sites directly involved in the virus-host coevolution [42]. SARS-CoV-2 is no different and is quickly adapting to its new human host, and the first major step in this direction was a D614G mutation in the spike protein. In a short time, multiple structural and biochemical studies demonstrated that the virus harboring D614G mutation is more infectious than the Wuhan-Hu-1 isolate [43–46]. Since then, several genetic variants of SARS-CoV-2 have descended from it and spread around the world. The SARS-CoV-2 Interagency Group (SIG) in collaboration with the Centers for Disease Control and Prevention (CDC) has categorized 4 classes of SARS-CoV-2 variants, namely, variants being monitored (VBMs), variants of interest (VOIs), variants of concern (VOCs), and variants of high consequence (VOHCs) [25]. There are currently 10 VBMs (Alpha (B.1.1.7) [47,48], Beta (B.1.351) [49], Epsilon (B.1.427) [25], Eta (B.1.525) [25], Iota (B.1.526) [25], Kappa (B.1.617.1) [25], Zeta (P.2) [25], Mu (B.1.621) [25], and Gamma (P.1) [25,50]) and 2 VOCs (Delta (B.1.612.2) [24] and Omicron (B.1.1.529) [25]) as of December 19, 2021. In the following section, we discuss the impacts of key mutations of selected VBMs and VOCs in the context of the structure and function of the spike protein.

### 2.1. Alpha

**Fig 2:**
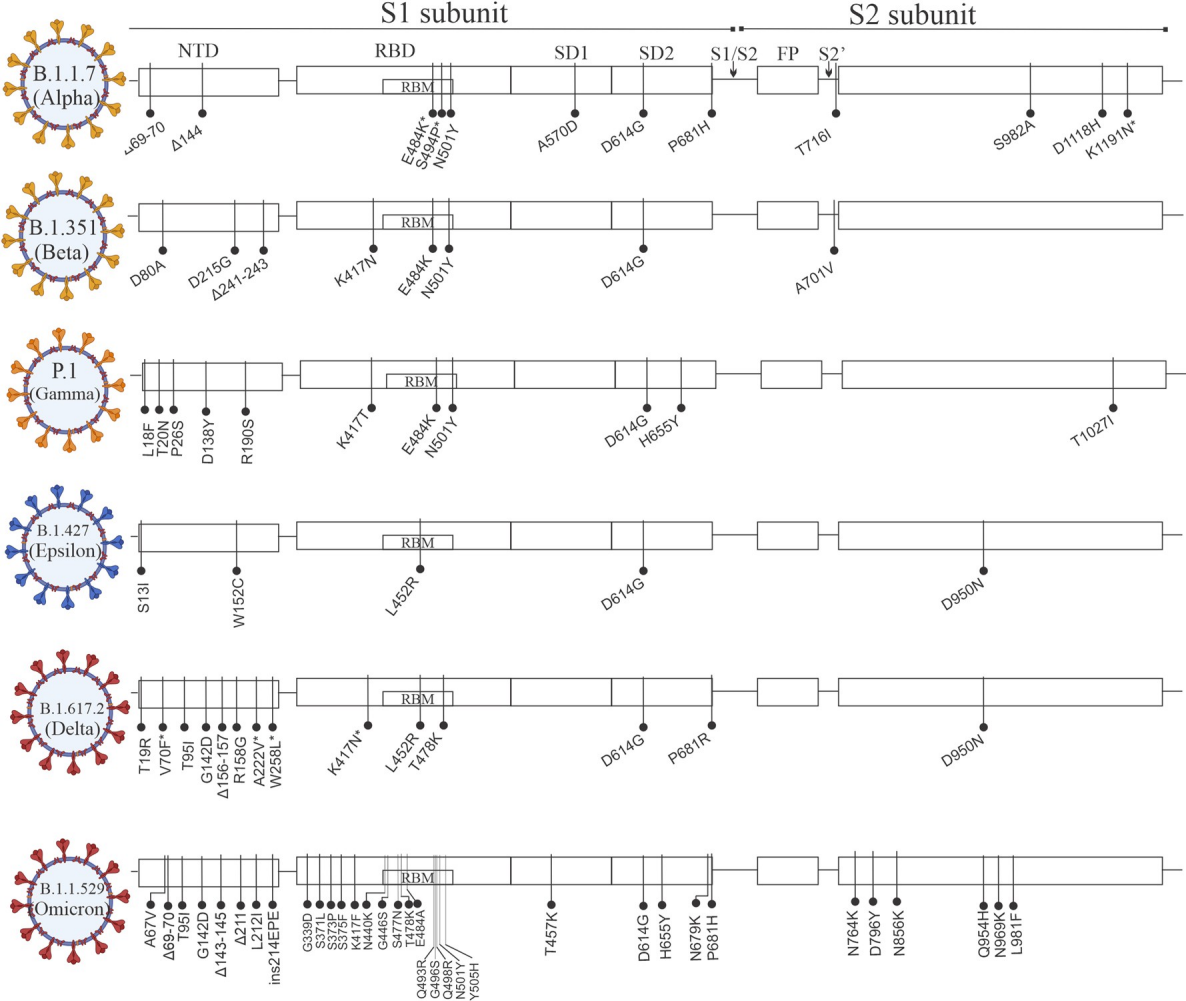
Schematic overview of the SARS-CoV-2 variants. The variant being monitored B.1.1.7 (Alpha), B.1.351 (Beta), P1 (Gamma), and B.1.427 (Epsilon) and the variant of concern B.1.617.2 (Delta) and B.1.1.529 (Omicron) showing amino acid modifications in comparison to the ancestral Wuhan-Hu-1 sequence (NC_045512.2). FP, fusion peptide; NTD, N-terminal domain; RBD, receptor-binding domain; RBM, receptor-binding motif; SARS-CoV-2, Severe Acute Respiratory Syndrome Coronavirus 2.

(B.1.1.7 lineage): This variant was first reported in the UK in September 2020, and since then, several countries have reported variants of this lineage. The characteristic features of this variant include Δ69/70 and Δ144 in the NTD and N501Y mutation in the RBD (Fig 2). Although the Δ69/70 in the NTD does not confer resistance to mAbs, it increases virus infectivity driven by higher levels of spike incorporation into virions. The Alpha variant induces faster syncytium formation that results in enhanced cell–cell fusion than the D614G variant [51]. Though N501 is one of the key residues that increases the RBD-ACE2 binding affinity [32,52], but the N501Y mutation has been shown to improve the binding affinity in vitro by hydrophobic interactions of the RBD Y501 with ACE2 Y41, and favorable cation–π stacking interactions with ACE2 K353 [21,53,54]. Overall, the mutations in the B.1.1.7 variant did not cause a major structural rearrangement in the RBD and NTD, which is apparent from a minimal change in the sensitivity of the Alpha variant to several potently neutralizing antibodies (nAbs). Among them, C63C8, C12A2, G32B6, C63C7 and C83B6, C81D6 are RBD and NTD targeting antibodies, respectively [54]. Taken together, it is concluded that the higher fusogenic potential [51] and improved fitness for replication in the upper airway [53] of the B.1.1.7 virus contribute to the higher mortality [55] and enhanced transmissibility [48,53]. Currently approved vaccines of Pfizer and AstraZeneca work against this variant with long-term efficacy comparable to that of D614G [56].

### 2.2. Beta

(B.1.351): This variant was first reported in Nelson Mandela Bay, South Africa, and since then, it has been reported in several countries including the USA. This variant has multiple mutations in the spike protein, including K417N, E484K, and N501Y, which were also seen in some of the Alpha variants; and amino acid deletions at 241–243 position (Fig 2). The Δ241–243 in the NTD (and Δ144 in B.1.1.7), N501Y, and E484K mutations are associated with reduced binding of certain nAbs and human serum antibodies [27,28,37,38,41,57–60]. The triple-residue deletion in the NTD causes a shift of nearby loops designated as N3 (residues 144 to 155) and N5 (residues 246 to 260), which form part of the NTD neutralizing epitope [61] (see section 4.5). The E484K mutation results in increased electrostatic interactions at the RBD-ACE2 interface, and multiple studies have confirmed that the variants harboring E484K mutations in the RBD exhibit considerably stronger interactions to human ACE2 receptor [62–64], suggesting their contribution to a higher rate of transmission than previous variants (Fig 2). Recently, an in silico study has implicated weakening of the T and B cells interactions in the Beta variant, resulting in escape from immune surveillance [64]. Currently approved vaccines of Pfizer and AstraZeneca neutralize this variant with a reduced efficacy as compared to D614G and alpha [56].

### 2.3. Gamma

(P.1 lineage): This variant was first reported in Japan by 4 travelers from Brazil. This variant is a descendent of B.1.1.28 lineage. Though both Gamma and Beta variants possess similar mutations in the RBD (K417T/N, E484K, and N501Y), but the Gamma variant is surprisingly less resistant to naturally acquired or vaccine-induced antibodies than the Beta variant, suggesting that distantly located mutations appear to have an impact on neutralization [60]. For example, mAbs 5–24 and 4–8 (which target the same NTD-antigenic supersite as DH1050.1) lost neutralizing activity against B.1.351 but not P.1 [65]. Both Gama and Beta variants have multiple substitutions in the C-terminal of the spike protein with unknown significance (Fig 2). Currently approved vaccines work against this variant with rare events of breakthrough infections.

### 2.4. Delta

(B.1.617.2): The SARS-CoV-2 lineage B.1.617 comprises 3 main sublineages: B.1.617.1 (Kappa), B.1.617.2 (Delta), and B.1.617.3, which may have diverse spike mutations in the RBD and the NTD. The Kappa and Delta variants were first reported in India in December 2020 [25,66], and since then, the Delta variant has spread around the world. This variant is specified by 11 mutations: T19R, T95I, G142D, Δ156–157, R158G, L452R, T478K, D614G, P681R, and D950N in the spike protein (Fig 2). Of these mutations, it has been observed that the P681R mutation is highly conserved in this lineage that facilitates the furin cleavage more efficiently, which drives enhanced fusogenicity and pathogenicity [66–68]. Interestingly, the Alpha variant, too, has a missense mutation at the amino acid position 681 with a histidine residue (P681H), but, interestingly, the Delta variant is about 60% more transmissible than the Alpha variant [24]. It has been observed that several mutations in the RBDs of these variants are located within and at the periphery of the ACE2-binding surface, suggesting that the virus accumulates mutations to avoid recognition by antibodies while maintaining binding to ACE2. For example, the L452R mutation in the Delta variant is located at the periphery of the ACE2-binding surface, which impairs neutralization by antibodies [69]. Instead of E484K, the Delta variant contains a unique mutation at a neighboring location, T478K, which facilitates immune escape due to a reduction or loss of binding by Class 1 and certain Class 2 nAbs [70]. The Delta and Omicron variants contain multiple mutations/deletions in the NTD supersite (see section 3.5.), which have not been observed in other major variants (Fig 2). These alterations result in reshaping the antigenic surface of the NTD loop (amino acid position 140–156) and a change in the glycan shield around the antigenic supersite, as indicated by the structural analysis of the Delta variant spike protein [21]. These modifications develop a total escape for the NTD-specific nAbs isolated from convalescent patient sera, infected with early Wuhan-related strain, and a reduction in neutralization by vaccine-elicited sera [56].

SARS-CoV-2 vaccines based on the Wuhan-Hu-1 spike glycoprotein elicit a varied degrees of immune responses in individuals that have not previously been infected with SARS-CoV-2. With the Pfizer vaccine BNT162b2, it was observed that nAbs were low against the D614G variant and almost undetectable against the Alpha, Beta, and Delta variants. However, nAbs titres improved significantly after the second dose, and a 3-fold and 16-fold reduction in the neutralization titres were observed against the Delta and the Beta variants, respectively, when compared to the Alpha variant [66]. A similar trend was also noticed with the sera obtained from the AstraZeneca ChAdOx1 nCoV-19 vaccinated individuals [66]. Thus, both mRNA and adenoviral vector-based vaccines generate a neutralizing response that efficiently targets the SARS-CoV-2 variants only after the second dose.

### 2.5. Omicron

(B.1.1.529): This variant was first detected in Botswana and then in South Africa in November 2021. Omicron has at least 34 mutations in spike protein, of which 15 are in RBD, and 7 changes in the NTD, and 3 mutations close to the furin cleavage site [25] (Fig 2). Some of the mutations in the spike protein of Omicron are known to increase transmissibility, and preliminary reports indicate that it is even more transmissible than the Delta variant [71,72]. For example, N501Y increases binding to the ACE2 receptor, which could increase transmission, and the combination of N501Y and Q498R may increase binding affinity even further. P681H has already been shown to enhance spike cleavage in the Alpha and Delta variants (P681R), and the Omicron variant contains 3 mutations (N679K, H655Y, and P681H) close to the furin cleavage site [25,73]. Planas and colleagues, in a recent study, found that among 9 mAbs in clinical use or in development, 6 (such as LY-CoV555, LY-CoV016, REGN-10933, REGN-10987, Tixagevimab, and Regdanvimab) lost antiviral activity against Omicron, while ADG-20 and Sotrovimab were less affected by the mutations in the Omicron variant with a half maximal inhibitory concentration (IC_50_) of 1,114 and 391 ng/ml, respectively (Tables 1 and S1) [73]. They further observed that sera obtained from individuals receiving the Pfizer and AstraZeneca vaccines after 5 months of 2 doses displayed no antiviral activity against the Omicron variant [73]. However, Andrews and colleagues, in a recent study, reported that Pfizer-BioNTech vaccine retained approximately 37% of vaccine effectiveness after 15 weeks of 2 doses of Pfizer-BioNTech vaccine. Remarkably, the vaccine effectiveness increased to about 75% after third dose (also known as booster dose) of Pfizer-BioNTech vaccine [71]. Similar reports from Pfizer-BioNTech and Moderna press releases indicate that mRNA vaccines are still incredibly effective against the Omicron variant after the booster dose. Future studies with a large cohort of individuals and patients are needed to validate these findings, as well as to understand how different the Omicron is from the previously emerged SARS-CoV-2 variants.

**Table 1:** Efficacy of clinical mAbs, lineages, and canonical mutations in SARS-CoV2 VOCs. REGN-CoV (REGN10987/Imdevimab and REGN10933/Casirivimab) by Regeneron, VIR-7832 and VIR-7831 (Sotrovimab) by GSK and Vir biotechnology, and LY-CoV555 (Bamlanivimab) and LY-CoV016 (Etesevimab) by Eli Lilly and Company are some of the approved antibody cocktails for emergency use in mild-to-moderate COVID-19 patients. These antibodies were obtained from the people who got infected with SARS-CoV-2 during the first wave of the pandemic. These therapeutic antibody cocktails targeting noncompeting epitopes have varied degrees of neutralizing efficacies against VOCs. The color of highlighted text represents the levels of efficacy mAb retains against each VOC: green represents the mAb retains efficacy; light-green represents reduced efficacy; and orange represents that the mAb is ineffective. ADG-20 developed by Adagio binds to an epitope located in between the Class 1 and Class 4 sites.

Variant Name			Alpha	Beta	Gamma	Epsilon	Delta	Omicron
Current CDC Classification			VBM	VBM	VBM	VBM	VOC	VOC
**Lineage**			B.1.1.7	B.1.351	P.1 or 20J/501Y.V3	B.1.427 or B.1.429	B.1.617.2	B.1.1.529
**Canonical mutations**			N501Y, 69/70 deletion, P681H	K417N, E484K, N501Y	K417T, E484K, and N501Y	S13I, W152C, L452R, D614G	L452R, T478K, D614G and P681R	Listed in Note 1
**Clinical mAbs**	Bamlanivimab (LY-CoV555)	Class 2	Effective	Ineffective	Ineffective	Ineffective	Ineffective	Ineffective
	Etesevimab (LY-CoV016)	Class 1	Reduced	Ineffective	Ineffective	Effective	Effective	Ineffective
	Casirivimab (REGN10933)	Class 1	Effective	Reduced	Ineffective	Effective	Effective	Ineffective
	Imdevimab (REGN10987)	Class 3	Effective	Effective	Effective	Effective	Effective	Ineffective
	Sotrovimab (VIR-7831)	Class 3	Effective	Effective	Effective	Effective	Effective	Effective
	Adintrevimab (ADG-20)	Class 1 and 4	Effective	Effective	Effective	Unknown	Effective	Effective

CDC, Centers for Disease Control and Prevention; COVID-19, Coronavirus Disease 2019; mAb, monoclonal antibody; SARS-CoV-2, Severe Acute Respiratory Syndrome Coronavirus 2; VBM, variant being monitored; VOC, variant of concern.

## 3. Antibody responses to SARS-CoV-2 infection

**Fig 3:**
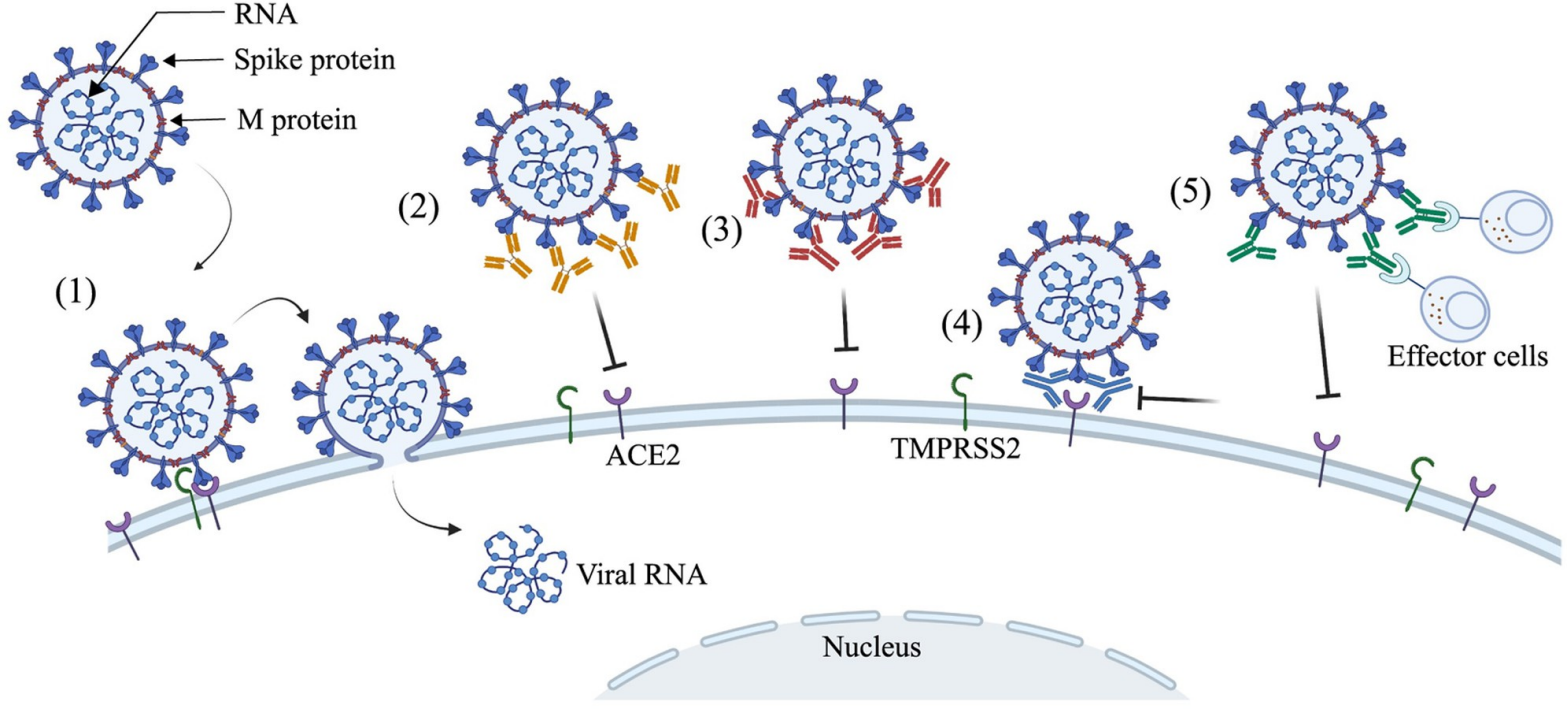
Antibody-mediated neutralization of envelope virus. SARS-CoV-2 virus entry into human cells is initiated by virus binding to the ACE2-cell surface receptors (point 1). The virus neutralization largely depends on the epitope targeted by antibodies. Some of the antibodies target the RBM (point 2) or NTD (point 3) or other regions of the spike protein, which can inhibit the virus spike protein and host ACE2–receptor interactions, and they are considered among the most potent nAbs. A few rare antibodies can inactive the fusion machinery by activating the premature fusion pathway, thus inhibiting the virus entry into the host cell (point 4). Some of the non-nAbs bind to the viral antigens and activate the Fc-mediated antibody effector functions for their killing or phagocytosis (point 5). The figure was prepared using BioRender. ACE2, angiotensin-converting enzyme 2; Fc, fragment crystallizable; nAb, neutralizing antibody; NTD, N-terminal domain; RBM, receptor-binding motif; SARS-CoV-2, Severe Acute Respiratory Syndrome Coronavirus 2.

The long-lasting antibody responses are the manifestation of coordinated interactions between the B and T cells within germinal centers of lymphoid organs. It is known that certain viral infections, such as smallpox, yellow fever, measles, and polio, confer long-lasting humoral and cell-mediated immunity, and most importantly, the virus-specific antibodies play a key role in providing long-term protection. However, after more than a year, the emergence of antigenically distinct SARS-CoV-2 variants has become a reality and a major concern for the effectiveness of currently used vaccines and mAb therapies. Antibodies prevent the pathology associated with productive infection mainly by 2 pathways: First, the Fab domain of an antibody binds specifically to a viral protein and blocks the ability of the virus to enter cells, a process referred to as neutralization. Second, the fragment crystallizable (Fc) portion of an IgG or IgM can induce cell lysis and death of virus-infected cells through a variety of immune effector mechanisms, including antibody-dependent cell-mediated cytotoxicity (ADCC), antibody-dependent cellular phagocytosis (ADCP), and antibody-mediated complement activation [74–76] (Fig 3).

In a cohort of 647 SARS-CoV-2–infected patients from Italy, Switzerland, and the USA, Piccoli and colleagues observed that the antibody responses were mainly directed toward the spike and nucleocapsid proteins. They observed that the nucleocapsid protein–specific antibodies were not neutralizing, and about 90% of the neutralizing activity was mediated by the RBD-specific antibodies. The remaining neutralizing activity may be accounted for antibodies elicited against the S2 subunit and the NTDs [77]. Of note, the fusion machinery of the S2 subunit contains several antigenic sites and is more conserved than the S1 subunit, thus an attractive target for eliciting broadly neutralizing antibodies (bnAbs). These findings indicate that the lower level of glycosylation, multiple conformations that expose cryptic sites, and higher accessibility on the viral surface are some of the reasons behind the enhanced immunodominance of the RBDs. To understand the antigenic anatomy of the SARS-CoV-2 spike protein, and the molecular mechanism of SARS-CoV-2 neutralization mediated by numerous mAbs, several laboratories performed biophysical and structural studies of distinct COVID-19 nAbs in complex with the trimeric spike protein [70,77–83]. This information has allowed the classification of these antibodies into different categories. In brief, Hastie and colleagues analyzed the 186 RBD-reactive mAbs using a high-throughput surface plasmon resonance technique that broadly grouped RBD-directed mAbs into 7 discrete communities and functionally relevant subclusters. They further mapped the footprints of 25 RBD-reactive mAbs by negative stain electron microscopy. Combining these biophysical and structural data together, the 7 communities were referred to as RBD-1 to RBD-3, RBD-4 to RBD-5, and RBD-6 to RBD-7, which are discussed in the next sections [80]. Tong and colleagues and Yuan and colleagues have also independently identified 7 and 3 major categories of the antibodies, respectively [81,83]. Here, in this review, we will discuss the classification of antibodies based on whether they bind an overlapping footprint with the ACE2 receptor and recognize an open or closed or both states of the RBD on the spike protein [70].

### 3.1. Class 1 neutralizing antibodies: Direct ACE2 competition by binding “up” RBDs

**Fig 4:**
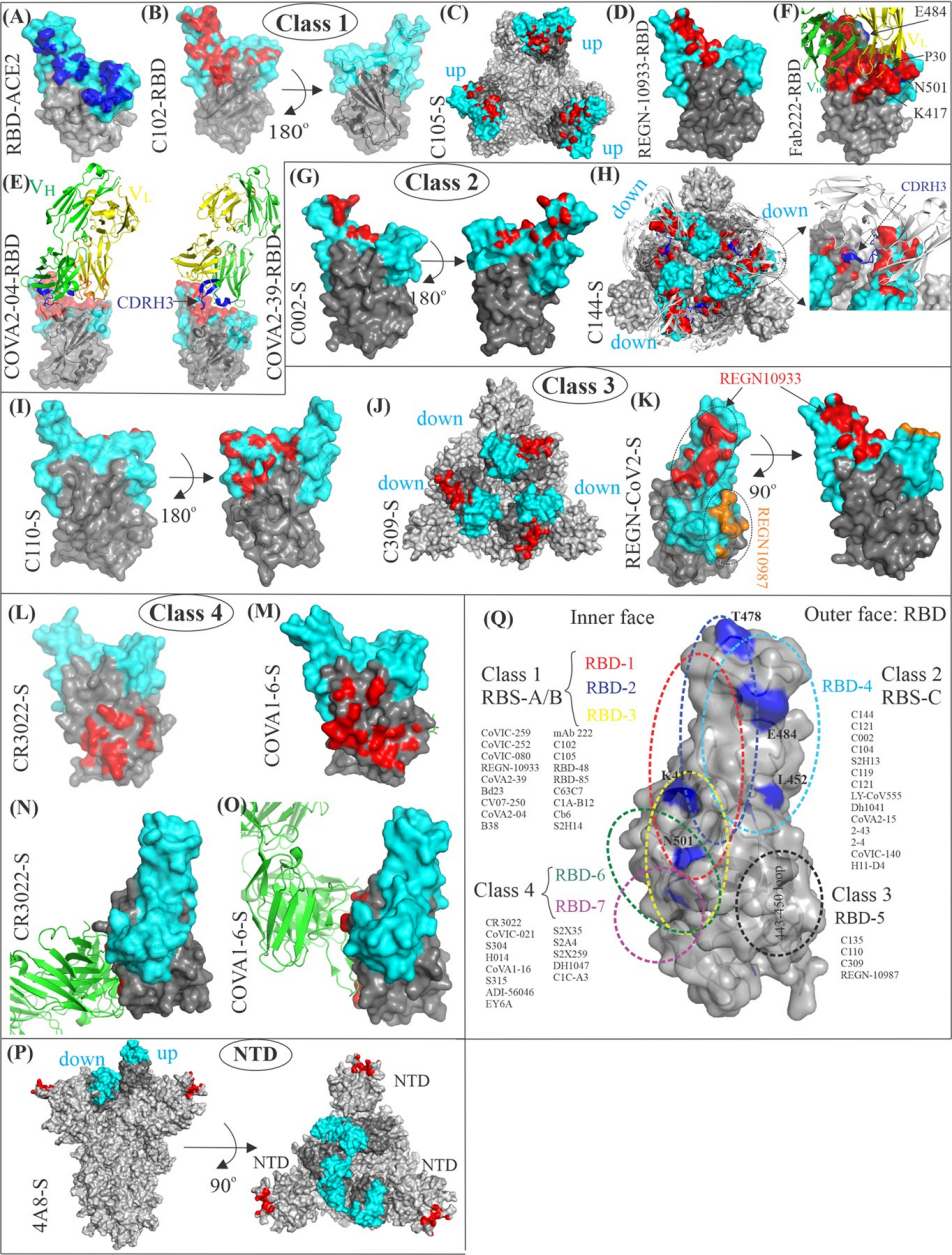
Structural analysis and classification of nAbs of the SARS-CoV-2 spike protein. (A) Structural footprints of ACE2 in blue on the RBM (cyan) (PDB: 6M0J). (B, C, D) The C102, C105, and REGN-10933 antibodies in complex with either isolated RBD or the spike trimer illustrate a conserved mode of binding to RBD in the up-conformation. Binding of C102 to the RBM on the RBD in red overlaps with the ACE2-binding site (PDB: 7K8M). (E) COVA2-39 (PDB: (7JMP) with a relatively longer CDRH3 loop binds the RBD in an alternative binding mode, as compared to the antibodies with a shorter CDRH3 loop, such as COVA2-04. (F) Fab222 binds RBD (PDB: 7NX6) with a relatively shorter CDRH3 loop. Residues K417, E484, and N501 are mutated in Alpha, Beta, and Gamma variants and are highlighted in red on the cyan ACE2-binding interface. (G) Structural footprints of a Class 2 antibody C002 on the RBD in red illustrates that the binding occurs toward the outer edge of the RBM (PDB: 7K8S). (H) The C144-spike protein complex structure revealed 3 C144 binding to a closed spike with all down-conformation RBDs (PDB: 7K90). The C144 epitope (red) spans between 2 adjacent RBDs on the surface of the trimeric spike. Close-up view of a quaternary epitope of C144 (red) bridging 2 adjacent monomers by the CDRH3 loop (blue). Class 1 and class 2 antibodies have significant overlap, but class 2 antibodies have fewer structural footprints on the ACE2-binding site at the RBM than class 1 antibodies (see B and G) (I) Structural footprints of a Class 3 antibody C110 on the RBD in red illustrates that C110 recognize a conserved epitope away from the receptor binding site and toward the outer face of the RBD. (J) The C309-spike protein structure illustrates that they bind outside of the ACE2-binding site. (PDB: 6WPS). (K) REGN-CoV2 is an antibody cocktail comprised of REGN10933 and REGN10987. REGN10933 blocks the ACE2-binding site like Class 1 antibodies, and REGN10987 sterically interferes with the ACE2 interactions like Class 3 antibodies. (L, M) Structural footprints of Class 4 antibodies CR3022 and COVA1-16 on the RBD in red, which illustrate that a conserved cryptic epitope accessible only in the up-conformation is recognized. The binding site of Class 4 antibodies is located away from the receptor binding site and toward the inner face of the RBD. (N, O) The CR3022 and COVA1-16 target a similar region on the RBD but with different angle, which explain their potency differences. (P) The structure of the 4A8-spike protein complex revealed that the NTD of each protomer is bound with 4A8 Fab. The structural footprints of 4A8 on the NTDs are shown in red. (Q) Summary of RBD-directed antibodies based on the binding pattern and competition profiles. The footprint residues on the RBD and NTD have been defined as those residues, which were within 4 Å of a Fab atom. ACE2, angiotensin-converting enzyme 2; nAb, neutralizing antibody; NTD, N-terminal domain; RBD, receptor-binding domain; RBM, receptor-binding motif; SARS-CoV-2, Severe Acute Respiratory Syndrome Coronavirus 2.

The large majority of Class 1 nAbs are encoded by the *VH3-53* or *VH3-63* gene segments, and their engagement with the ACE2-binding site on the RBDs is dominated by CDRH1 and CDRH2, whereas a shorter CDRH3 loop (less than 15 amino acids) makes fewer interactions with the ACE2-binding site on the RBDs [60,70,78,84]. The binding orientations of these antibodies are only compatible with the up-RBDs, and due to the complete occlusion of the ACE2-binding interface, no interactions with the down RBDs are allowed (Fig 4A and 4F). Antibodies from the RBD-1, RBD-2, and RBD-3 communities, described by Hastie and colleagues, require the up-RBD conformation for competing with the ACE2 receptor [80]. The footprint of RBD-1 antibodies, such as CoVIC-259 (EMD: 24335), largely overlaps with the RBM (Fig 1A and 1B), while the binding of CoVIC-252 (EMD: 24339), which is an RBD-2 antibody, is shifted from the center of the ACE2-binding site toward the receptor-binding ridge. RBD-Cluster 2a antibodies bind toward the inner face of the RBD, similar to REGN-10933 (PDB: 6XDG) [85] (Fig 4D and 4Q), and Cluster 2b binds toward the outer face of the RBD, similar to COVA2-39 antibodies (PDB: 7JMP) [86] (Fig 4E). Lastly, the RBD-3 mAbs, such as CoVIC-080 (EMD: 24346), bind toward the right shoulder of the RBD [80]. Piccoli and colleagues have defined the RBM targeting site by S2H14 as Ia, which largely overlaps with the ACE2-binding site and is only accessible in the up-RBD state [77]. Structural analysis of Class 1 (or RBD-1 through RBD-3) antibodies in complex with the spike protein indicate that they use 2 general binding modes, which depend on the CDRH3 loop length. For example, antibodies with a short CDRH3 length (approximately 10 amino acids) form the majority of Class I mAbs, such as COVA2-04 (7JMO), C102 (PDB: 7K8M) [70], and others (Fig 4B, 4E and 4F), while a small subset of antibodies, such as COVA2-39 (PDB: (7JMP) [86], BD23 (7BYR) [87], and CV07-250 (6XKQ) [88] adopt an alternative binding mode to fit a relatively longer CDRH3 loop (approximately 15 amino acids) into a restricted space between the antibody and the RBDs. This demonstrates the versatility in targeting the RBDs of SARS-CoV-2. Although both COVA2-04 and COVA2-39 antibodies block the RBD-binding site and bind with similar affinities (Kd of 40 and 21 nM, respectively), viral neutralization assays indicate that COVA2-39 is more potent than COVA2-04 in neutralizing SARS-CoV-2 (IC_50_ values of 0.036 and 0.22 mg/ml, respectively). One of the reasons behind this disparity could be attributed to the different binding modes adopted by these antibodies [86] (Fig 4E). Dejnirattisai and colleagues recently discovered a cross-reactive antibody, mAb 222, which can neutralize all 3 B.1.1.7, B.1.351, and P.1 variants despite its interactions with 2 of the ACE2-binding site mutations (K417N/T and N501Y) [60] (Fig 4F). The crystal structure of the RBD-Fab 222 complex (PDB: 7NX6) indicates that the CDRH1 and CDRH2 weakly interact with K417N/T of the RBD, and CDRL1 interacts even stronger with N501Y of the RBD due to a rare somatic mutation (Proline 30) that lead to the formation of a *cis*-amide bond between proline and tyrosine [89,60] (Fig 4F). Interestingly, the chimeric antibodies derived from the combination of the light chain of mAb 222 and the heavy chain of other weakly or nonneutralization VH3-53 antibodies, such as mAbs 150, 158, 175, and 269, were able to restore neutralization potency [60]. The Class 1 antibodies include B38 (PDB: 7BZ5) [90], C102 (PDB: 7K8M) [70], C105 [PDB: 6XCM (2 RBDs “up”), 6XCN (3 RBDs “up”) [79]], S2H14 (EMD: 22508) [77], RBD-48 [66], RBD-85 [66], C63C7 [54], C1A-B12 (PDB: 7KFV) [91], mAb 222 (PDB: 7NX6) [60], REGN-10933 (PDB: 6XDG) [85], CB6 (7C01) [92], and COVA2-04 (PDB: 7JMO) among many others.

### 3.2. Class 2 neutralizing antibodies: Direct ACE2 competition by binding “up” and “down” RBDs

Structures of Class 2 (or RBD-4) antibodies in complex with the spike protein have revealed that they recognize RBDs in the “up” and “down” conformations by binding toward the outer edge of the RBM, and their epitopes overlap the ACE2-binding site but less than Class 1 members (Fig 4B and 4G). The Class 2 antibodies are encoded by a variety of the *VH3* gene segments and are characterized by a longer CDRH3 loop of 25 amino acids [70,84]. The C144-S complex structure (PDB: 7K90) revealed 3 C144 Fabs binding to 3 “down” RBDs on the spike protein, and likely locking the spike protein in a prefusion conformation by inserting the CDHR3 loop between 2 adjacent RBDs (Fig 4H). Thus, it is plausible to speculate that the C144-mediated sealing of the spike protein in the prefusion conformation might prevent breathing in the RBDs that potentially restrict them to flip to the up-conformation, in which the spike protein can bind to the ACE2 receptor. Due to these characteristics, C144 is a potent nAb with an IC50 of <10 ng/ml and is in clinical development for prophylaxis [93].

However, contrary to C144 binding, the structures of C002 (encoded by VH3-30 and VK1-39 gene segment; 17 amino acids long CDRH3 loop) and C121 (encoded by VH1-2 and VL2-23 gene segment; 23 amino acids long CDRH3 loop) in complex with the spike protein showed 2 modes of interactions, where they appear to bind simultaneously to 2 RBDs, from adjacent protomers, in both “up” and “down” conformations by forming a quaternary epitope [70]. In brief, along the lines of C144, the C002 and C121 RBD epitopes are concentrated largely on the receptor-binding ridge through interactions with light-chain CDR loops and with the RBD flat face engaged with heavy-chain CDR loops (Fig 4G and 4H) [70].

Regardless of the orientations in the context of binding the spike protein, the C144, C121, and C002 epitopes overlap with the ACE2-binding site, suggesting that the neutralization mechanism involves direct competition with the ACE2 receptor. These antibodies include C002 (PDB: 7K8S) [70], C104 (PDB: 7K8U) [70], S2H13 (PDB: 7JV2) [77], C119 (PDB: 7K8U) [70], C121 (PDB: 7K8X) [70], LY-CoV555 (PDB: 7KMG), DH1041 (7LAA), COVA2-15 (EMD-22061) [82], 2–43 (EMD-22275) [94], 2–4 (EMD-22156, PDB: 6XEY) [94], CoVIC-140 (EMD-24383) [80], and H11-D4 (PDB: 6YZ5) among many others. Piccoli and colleagues have defined the RBM targeting site by S2H13 as Ib, which partially overlaps with the ACE2-binding site and remains accessible in both open and closed states of the spike protein [77].

### 3.3. Class 3 neutralizing antibodies: Bind outside the direct RBD-ACE2 interface in both “up” and “down” RBDs

Class 1 and 2 antibodies inhibit SARS-CoV-2 virus entry by physically blocking the ACE2-binding motif; however, there are other conserved regions on the RBD that are away from the receptor binding site but are targeted by antibodies for achieving virus neutralization. Class 3 antibodies (or RBD-5) recognize conserved epitopes located on the opposite side of the receptor-binding motif, including the 443–450 loop, which is accessible in both the up and down RBD conformations (Figs 1C and 4I). Several antibodies, such as C135, C110, and C309, bind the RBDs in the up- as well as in the down-conformations (Fig 4J), and they mediate neutralization either by steric hindrance, locking and cross-linking the trimeric spike protein, or by Fc-mediated antibody effector functions [95]. The structures of Class 3 antibodies in complex with the spike protein exhibit that the cross-linking of adjacent RBDs is possible in certain cases [80]. Since these antibodies bind outside the receptor-binding site, therefore, they leave open the possibility of being an ideal partner with Class 1 or 2 antibodies in designing effective therapeutic cocktail therapy. REGN10987 and REGN10933 represent such a pair of antibodies, where both antibodies can simultaneously bind at different epitopes on the RBD. In brief, REGN10987 binds at the side of the receptor-binding motif, including the 443–450 loop, like C110, and REGN10933 binding overlap with the ACE2-binding site [85] (Fig 4K). Preclinical studies have confirmed that a cocktail of REGN10987 and REGN10933, referred to as REGN-COV2, shows enhanced clearance of virus and suppresses the rapid emergence of antibody resistance [31]. S309 is a class 3 antibody that was screened from the blood sample of a SARS-CoV–infected patient. It binds to a conserved epitope that has been termed by Piccoli and colleagues as site IV [77]. It potently neutralizes SARS-CoV, SARS-CoV-2, and SARS-related coronavirus WIV-1 by binding to a conserved epitope containing a glycan (N343 in SARS-CoV-2) on the RBD. S309 has shown increased neutralization potencies in combination with weakly nAbs and additionally exhibited that antibody cocktail reduces the generation of escape mutants [95]. VIR-7831 and VIR-7832 are dual-action mAbs in clinical use, which are derived from the S309 antibody [96]. REGN-COV2 and VIR-7831/VIR-7832 both have received emergency use authorization (EUA) for the treatment of mild-to-moderate COVID-19 patients. Other mAbs and medical countermeasures, which have been thus far given EUA and are under development, are listed in Tables 1 and S1.

### 3.4. Class 4 neutralizing antibodies: Bind to a cryptic epitope in the “up” RBDs

RBD-6 and RBD-7 mAbs constitute the Class 4 antibodies [80]. These antibodies require at least 2 RBDs to be in the up-conformation for targeting a cryptic epitope that is buried in the down-conformation and resides outside the ACE2-binding site (Fig 4L, 4M, 4N and 4O). The RBD-6 and RBD-7a antibodies sterically block the SARS-CoV-2 and ACE2 receptor interactions but not RBD-7b and RBD-7c. Antibodies such as CR3022 (PDB: 6W41) [97] and CoVIC-021 [80] are representative of RBD-7b and RBD-7c clusters and both demonstrate poor pseudovirus neutralization [80], suggesting indirectly that the screening of RBD-6 and RBD-7a communities that sterically block the RBD-ACE2 interactions might lead to the identification of nAbs against SARS-CoV-2. S304. H014 and COVA1-16 are examples of such antibodies [77,83,98,99]. The CR3022 (PDB: 6W41), S304 (PDB: 7JW0) [77], and S315 [77] antibodies are weakly neutralizing cross-reactive antibodies that were isolated from individuals infected with SARS-CoV [97], and H014 was isolated from a transgenic mouse [100]. The crystal structure of the CR3022-RBD complex revealed a conserved cryptic site that is only accessible when at least 2 RBDs are in the up-conformation [100] (Fig 4O and 4P). The site is found to be a target of other cross-nAbs against SARS-CoV-2, such as H014 (EMD: 30326) [98], COVA1-16 (PDB: 7JMW) [99], ADI-56046 [101], and EY6A (6ZER) [102]. Among them, H014 and COVA1-16 achieve high neutralizing activity by targeting the CR3022 binding site with a different angle, which effectively inhibit the binding of the trimeric spike protein to ACE2 [83,98,99] (Fig 4N and 4O). The binding epitopes of other mAbs, such as S2A4 (PDB: 7JVC, EMD: 22506), S2X35 (EMD: 22516), and S304 (PDB: 7JW0) [77], are also increasingly away from the ACE2-binding site and were termed by Piccoli and colleagues as site IIa, IIb, and IIC [77]. S2X259 (PDB: 7RA8 and 7RAL) [103] and DH1047 [104] are other nAbs of Class 4. Recently, Nabel and colleagues identified a mAb, C1C-A3, which can bind various SARS-CoV-2 variants, including Beta, Gamma, Delta AY.2, and Epsilon, by binding to the conserved RBD core that overlaps significantly with CR3022 epitope. CAIC-A3, similar to many Class 4 antibodies, shows high neutralizing activity against the SARS-CoV-2 variants by sterically interfering with ACE2 binding [105]. Of note, CR3022 only neutralizes SARS-CoV, not SARS-CoV-2, and C1C-A3 neutralizes SARS-CoV-2, not SARS-CoV. Thus, antibodies of this class can also be used in combination with other antibodies, which target the ACE2-binding site for therapeutic usage.

### 3.5. Neutralizing antibodies that target the N-terminal domain of the RBD

Although the RBD is an immunodominant part of the S protein, a small fraction of mAbs has been found binding to the NTDs, and a few of them exhibited neutralizing abilities against SARS-CoV-2 [61,94,106–108]. Chi and colleagues isolated such an antibody, named 4A8, which does not block the interactions between ACE2 and S protein but exhibits high neutralization potency against the SARS-CoV-2 virus. The 4A8 Fab binding stabilizes the NTD of the S protein that facilitated the building of 5 NTD loops, which are designated as N1 (residues 14 to 26), N2 (residues 67 to 79), N3 (residues 141 to 156), N4 (residues 177 to 186), and N5 (residues 246 to 260). The cryo-EM structure revealed that the NTD of each protomer is bound with 4A8 Fab (PDB: 7C2L) (Fig 4R), where 4A8 heavy chain mostly participates in binding with the N3 and N5 loops residues in the NTD (Y145, H146, K147, K150, W152, R246, and W258) of the spike protein [61]. DH1048 (EMD: 22936), DH1049 (EMD: 22942), DH1050.1 (EMD: 22944 and 23277, PDB: 7LCN), DH1050.2 (EMD: 22945), and DH1051 (EMD: 22946) are other NTD-specific nAbs [109], all of which target the same epitope as 4A8. Suryadevara and colleagues found 2 NTD-targeting antibodies from COVID-19 convalescent plasma, COV2-2676 and COV2-2489, which neutralize SARS-CoV-2 by inhibiting a post-attachment step in the infection cycle [106]. Interestingly, Fab fragments of these antibodies could not neutralize infection but required either intact IgG or F(ab)2 version for optimal therapeutic protection, suggesting that a larger size of antibodies either sterically inhibit functional interactions crucial for the entry process or Fc effector functions are also required for optimal protection or both.

Structural and biophysical analysis of many nAbs and non-nAbs in complex with the spike protein has led to the identification of an antigenic supersite on the NTD, which comprised of residues 14 to 20, 140 to 158, and 245 to 264 [107]. It was found that nearly all nAbs including 4A8 (PDB: 7C2L) [61], FC05 [110], CM25 [108], C12C9 [61], C83B6 [61], COVA1-22 (EMD-22062) [82], 4–8 (EMD-22158) [94], COV2-2676, and COV2-2489 [106] target the NTD supersite, suggesting an example of the convergent mode of binding for diverse antibodies to the same binding site. Other antibodies, such as 2–17, 5–24, 2–51, 5–7, 4–18, 1–87, 4–19, and 1–68 neutralizes the live SARS-CoV-2 virus with IC_50_ values of 0.007, 0.008, 0.007, 0.033, 0.020, 0.086, 0.109, and 0.014 μg/ml by binding to the NTDs at unknown locations [94]. Hastie and colleagues have identified 4 NTD-specific mAbs and grouped them NTD-1 through NTD-3. CoVIC-247 (EMD-24355) is an NTD-1 antibody that binds the NTD N-terminus and residue Y144, and its footprints overlap with that of 4A8 Fab [61] and other supersite binders [107,111]. The NTD-2 antibody (CoVIC-245, EMD-24360) interacts with Y144, H69, V70, W152, and G261 residues of the NTD, all of which are either deleted or mutated in emerging variants. CoVIC-020 (EMD-24356) is an NTD-3 mAb that binds to a novel epitope containing residue W152. These NTD-specific mAbs display a decreased or total loss of neutralization for one or more of the NTD-located deletions (Δ69/70, ΔY144, Δ157–158, and Δ242–244) found in SARS-CoV-2 variants [80].

Although the molecular mechanism of NTD-specific antibodies mediated neutralization is still not fully understood, it has been observed that they inhibit cell–cell fusion and activate effector functions to protect Syrian hamsters from SARS-CoV-2 challenge [107]. Several naturally occurring variants including the Alpha, Beta, and Gamma variants exhibit mutations in the NTD supersite, and these substitutions completely abolish the neutralizing activities of various NTD-specific nAbs [21,38,65,107]. This is one of the reasons that the RBD continues to be the main route for the formation of therapeutic antibodies (Tables 1 and S1).

## 4. Spike mutations and immune escape

**Fig 5:**
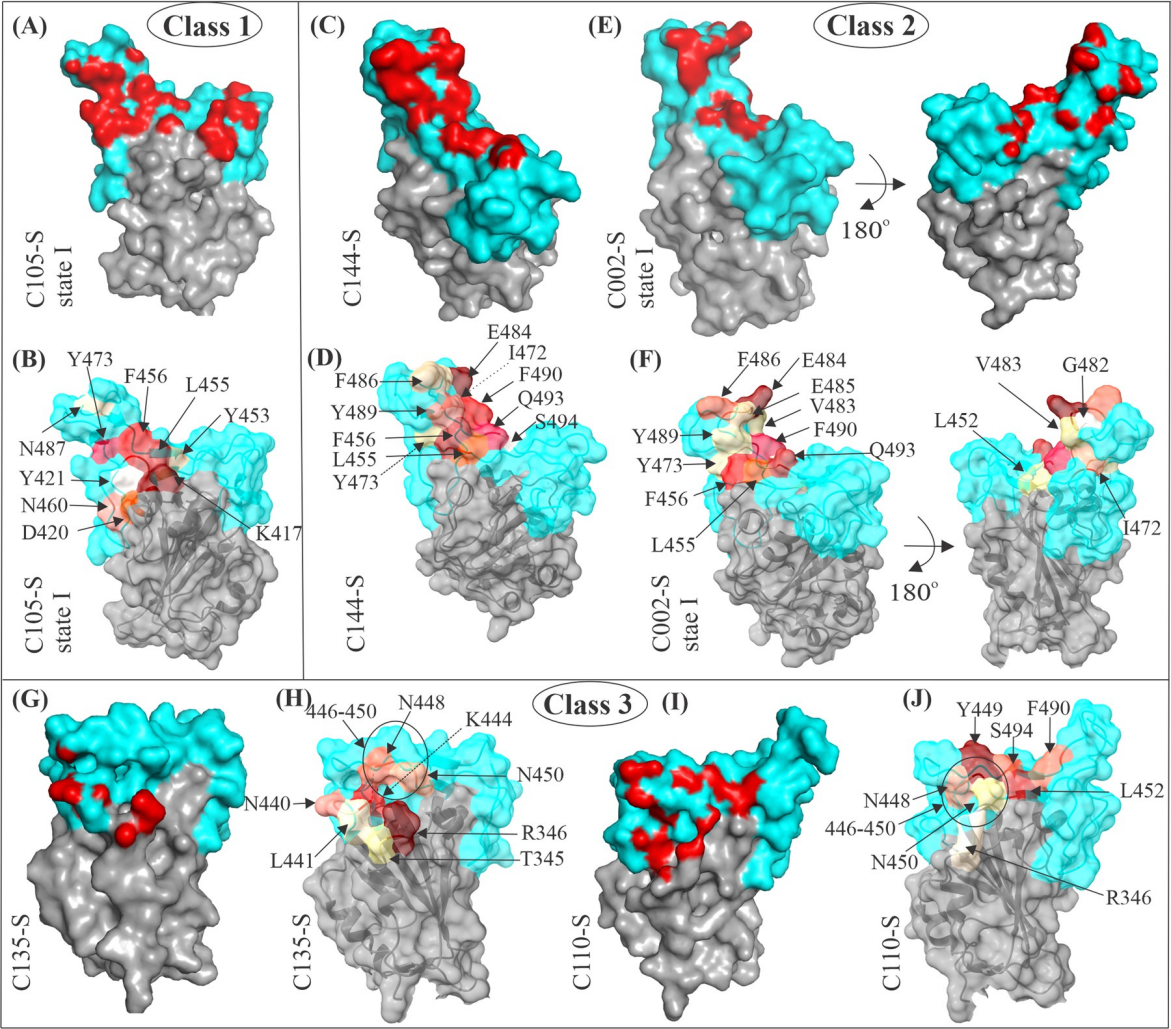
SARS-CoV-2 RBD mutations that escape antibody binding. Total escape at each site was measured using a yeast library screening followed by deep sequencing for distinct RBD antibodies (https://jbloomlab.github.io/SARS2_RBD_Ab_escape_maps/). The escape map identifies mutations that escape antibody binding and were mapped on the RBD-Fab structures. The red indicates the site with maximum and white with minimum resistance for antibody binding. (A, C, E, G, I) The structural footprint of C105, C144, C002, C135, and C110 antibodies on the RBM is in red. (B, D, F, H, J) Mapping of the SARS-CoV-2 virus escapes for different antibodies. Mutations that escape antibody binding were broadly located at their binding site in the RBD (see A-B, C-D, E-F, G-H, and I-J pairs). The footprint residues on the RBD have been defined as those residues, which were within 4 Å of a Fab atom. RBD, receptor-binding domain; SARS-CoV-2, Severe Acute Respiratory Syndrome Coronavirus 2.

The SARS-CoV-2 virus is continuously undergoing antigenic evolution driven by positive selection for mutations in the spike protein, particularly in the RBD. Antibodies generated either by vaccination or natural infection have been rendered ineffective by some of these variants that are selected in infected individuals. The atomic structures of the spike protein and nAbs complexes undoubtedly define the protein–protein interaction interfaces that are known as “structural epitope”; however, they do not define sites where mutations will affect antibody binding and neutralization. These sites are often referred to as “functional binding epitopes” and are usually smaller than the “structural epitopes” [26,112,113]. Several laboratories have generated functional maps of antigen-binding sites experimentally by different approaches but with the same goal [27–30,33,34,114]. In brief, in the yeast display deep mutational approach, yeast libraries expressing all possible amino acids substitutions in the RBD are incubated with either mAbs or polyclonal plasma, and then antibody escape cells are selected using fluorescence-activated cell sorting followed by deep sequencing to compute the escape fraction for each mutation. In other methods, a replication-competent chimeric vesicular stomatitis virus (VSV) encoding the SARS-CoV-2 spike protein is passaged in the presence of nAbs to select escape variants for each of the antibodies [30,33,34,114]. Weisblum and colleagues passaged rVSV/SARS-CoV-2 in presence of C121, C135, and C144 mAbs. In the case of C121, C135, and C144 antibodies, mutations E484K and Q493K/R, R346K/S/L and N440K, and E484 and Q493 were observed, respectively [30]. Greaney and colleagues used a yeast display system to gain a broad picture of viral escape by comprehensively mapping 22 different nAbs and polyclonal plasma targeting the RBD epitopes as discussed in the previous section [28]. The mutational antigenic profiling provides a comprehensive mutation-level scan of the functional binding epitopes between the RBD and nAbs. A comparison of the overlap between the structural epitopes and functional epitopes of the RBD-nAbs complexes lead to the following conclusion: Mutations that escape antibody binding are broadly located in or near the binding site on the RBD, and antibodies within the same class are escaped by mutations at similar sites in the RBD [28] (Fig 5). For example, neutralization by RBD-Cluster 2a antibodies is primarily affected by the K417N but hardly by the E484K mutation, and RBD-Cluster 2b antibodies are heavily affected by the E484K but less by the K417N mutation. However, RBD-3 antibodies are affected by both N501T/Y and E484K mutations [80]. Although class 1 and class 2 antibodies have significant overlap on their structural footprints (Fig 4B and 4J), escape mutants at K417, D420, N460, and S475 sites were observed for class 1 but not for class 2 antibodies. On the other hand, class 2 antibodies but not class 1 were escaped by mutations at sites L452R, E484, E490, and Q493, suggesting a functional diversity of antibodies despite having an overlap in their binding footprints (Fig 5A–5D). Class 3 antibodies were found to be escaped by mutations at R346, K444, and G446-450 sites [28] (Fig 5E and 5F). Of note, the antibody escape sites for HIV envelope protein were largely found outside the structural contact site, suggesting that an alteration of noncontact sites can also affect antibody sensitivity [26]. Notably, in the yeast display experiments, the isolated RBD was used; hence, they do not report comprehensively on the escape mutations at the quaternary binding sites on a trimeric spike protein. Overall, a remarkable match is observed between the antibody escape map and antibody binding footprints on the RBD (Fig 5A, 5B, 5C, 5D, 5E, 5F, 5G, 5H, 5I and 5J pairs), with a few exceptions. Interestingly, these artificially generated antibody escape mutations are present in the naturally emerging virus populations. In particular, E484K mutation most strongly escapes the class 2 antibodies, including class 1 antibodies in certain cases, and also exists in the Beta, Gamma, Zeta (P.2), and B.1526 variants [25]. Of these, Beta and Gamma also harbor class 1 antibody escape mutation, K417N or K417T, respectively. The class 3 escape mutations are found in very few variants, one example being the L452R mutation present in the Epsilon (B.1.427/429) and variants of B.1.617 lineage. However, no viral variant so far has emerged with those mutations that escape all 3 antibody classes. Therefore, close monitoring of SARS-CoV-2 sequences is warranted because the development of a strain that can escape all 4 antibody classes would be a big burden to the ongoing public health and vaccine development efforts.

## 5. Conclusions

Francis Crick famously said, “if you want to understand the function, study structure”. In the last more than a year, an unprecedented number of structures of the SARS-CoV-2 spike protein, including its variants, in complex with numerous mAbs have been determined to understand the molecular mechanism of antibody-mediated neutralization of SARS-CoV-2. The structural analyses of these complexes provide a comprehensive map of nAb epitopes on the spike protein. However, these analyses do not directly measure how/which mutations will escape from antibody binding. Going forward, mutations in the RBD that escape antibody binding were identified by different methods [27,28,30,32–34,114]. An analysis between the structural contact sites and escape maps suggested that these maps complement each other; therefore, comprehensive knowledge of immunodominant epitopes on the spike protein and escape mutations would aid efforts to understand viral evolution and rational design of antibody therapeutics, vaccines, and other countermeasures (Tables 1 and S1).

Antibodies that were isolated from the early COVID-19 phase were found to have a relatively low number of somatic mutations. However, it has been recently demonstrated that these antibodies show affinity maturation by somatic hypermutation in the lymphoid germinal centers, which empower them for increased binding affinity and neutralization potency for SARS-CoV-2 [84]. For example, C051 and C052 exhibit about 4-fold higher somatic hypermutation than the clonally related class 2 C144 antibody, and mutations in the RBD at L455, F456, F490, Q493, and S494 do not confer resistance anymore. These antibodies display an increased binding affinity for the RBD with Q493 mutation as compared to C144 but show resistance in binding to the RBD with E484K mutation [84]. It is important to note here that most of the affinity matured antibodies of class 2 were unable to neutralize SARS-CoV-2 pseudovirus with an E484K mutation alone or in combination with K417N and N501Y substitutions (B1.351 VOC) [84]. Overall, these results indicate that serological immunity is evolving to fight against the continuously evolving SARS-CoV-2 virus [84,115].

**Fig 6:**
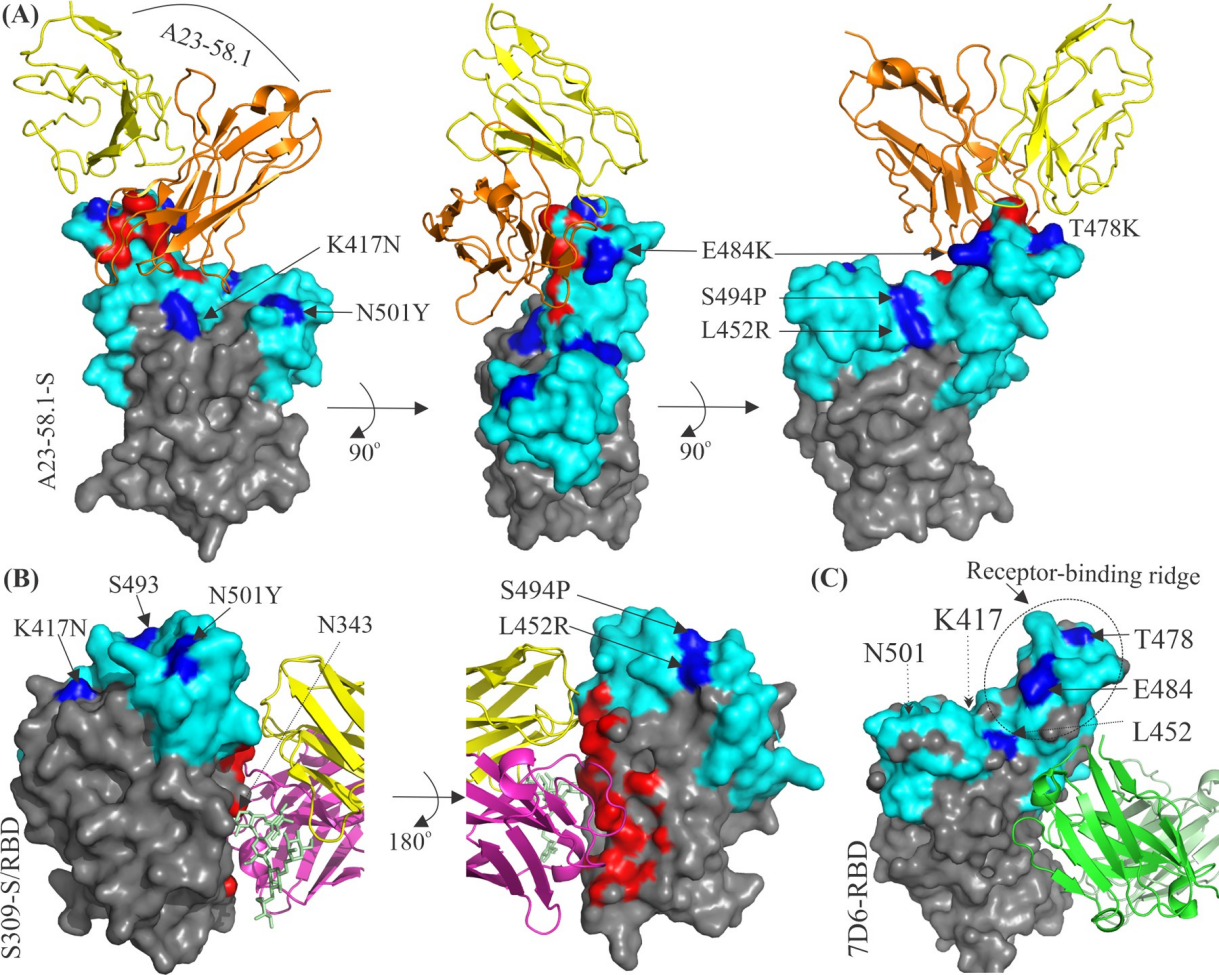
Structural analysis of bnAbs binding to the RBD. The epitope surface of the A23-58.1 antibody is shown in red. A23-58.1 targets the supersite with minimal contacts to major mutational hotspots (K417, L452R, E484, S494P, and N501) of current VOCs, which are shown in blue. The binding mode of A23-58.1 is very similar to those of class 1 antibodies. The CDR H3 of A23-58.1 contains 14 residues and can only bind an RBD in the up-conformation (PDB: 7LRT). The RBD is shown in gray containing the RBM in cyan at their top. (B, C) S309 is a class 3 antibody while 7D6 binds proximal to the S309 epitope. S309 recognizes an epitope containing a glycan in white (N343 in SARS-CoV-2). 7D6 binds a novel cryptic site located behind the receptor-binding ridge of the RBD that faces toward the NTD. The S309 and 7D6 binding site residues are conserved within the *Sarbecovirus* subgenus. Both antibodies are resistant to mutations that emerged in the SARS-CoV-2 variants. The footprint residues on the RBD have been defined as those residues, which were within 4 Å of a Fab atom. bnAb, broadly neutralizing antibody; NTD, N-terminal domain; RBD, receptor-binding domain; RBM, receptor-binding motif; SARS-CoV-2, Severe Acute Respiratory Syndrome Coronavirus 2; VOC, variant of concern.

SARS-CoV-2 will continue to evolve, and it is nearly impossible to predict future variants; therefore, identification of bnAbs is a pressing need of the hour due to their strength of neutralizing multiple variants of a virus by recognizing evolutionarily conserved epitope. In this direction, the RBD-targeting antibodies, A23-58.1 and B1-182.1, were identified by Wang and colleagues, which neutralize multiple SARS-CoV-2 variants, including the B.1.1.7, B.1.351, P.1, and B.1.617 variants at subnanomolar concentration [116]. They bind to an invariant region of the RBD tip that is offset from major mutational hotspots (K417, E484, and N501), which increases their breadth and potency (Fig 6). These mutations are the major determinants conferring resistance in the B.1.1.7, B.1.351, P.1, and B.1.617 variants (Fig 2). The mAb 222 antibody is another notable example that binds both P.1 (K417T, E484K, and N501Y) and the ancestral Wuhan-Hu-1 RBD with similar affinities despite differences in the binding site. Notably, mAb 222, a Class 1 antibody, can neutralizes all 3 B.1.1.7, B.1.351, and P.1 variants despite its interactions with 2 of the ACE2-binding site mutations [60] (Fig 4E). The RBD-Fab 222 structure (PDB: 7NX6) indicates that the CDRH1 and CDRH2 weakly interact with K417N/T of the RBD, and CDRL1 contains a proline (residue P30) due to a rare somatic mutation that forms a *cis*-amide bond [89] with N501Y of the RBD [60] (Fig 4E). The 7D6 is a recently isolated bnAb that binds away from the RBM and is not sensitive to currently circulating SARS-CoV-2 variants. The crystal structure shows that 7D6 binds a novel cryptic site located behind the receptor-binding ridge of the RBD that faces toward the NTD. The 7D6 antibody binding causes interference with the adjacent NTD and appears to destabilize the spike protein. Structural analysis has shown that the 7D6 binding site residues are nevertheless conserved within the *Sarbecovirus* subgenus and the 7D6-epitope location is distinct from those bound by most antibodies that are insensitive to the mutations [117] (Fig 6C). These antibodies are classified as bnAbs based on the data available and can be further modified and evaluated for their use in cocktail therapy.

In addition to bnAbs, broadly neutralizing nanobodies are also identified that are capable of neutralizing B.1.1.7 (Alpha), B.1.351 (Beta), and P.1 (Gamma) variants [118]. Xu and colleagues identified 2 groups of neutralizing nanobodies from llama and an engineered mouse (nanomouse). Nanomouse nanobodies, Nb12 and Nb30, target a conserved region on the RBD that is largely inaccessible to human antibodies and is located outside the ACE2-binding motif (PDB: 7MY3 and 7MY2), which explains why their binding is not affected by E484K (Beta and Gamma) or N501Y (Alpha) substitutions [118]. Going forward with the identification of more effective therapies against SARS-CoV-2, multiple laboratories have reported the development of bispecific antibodies by combining 2 antibodies that target nonoverlapping epitopes on the spike protein and multivalent nanobodies. These engineered molecules have been described to be highly potent in neutralizing the SARS-CoV-2 variants and suppressing mutational escape [119–122]. Alongside this, antibodies from the NTD and community RBD-1 to RBD-4 are an attractive choice for the therapeutic cocktails being more potent than others, but the data suggest that emerging SARS-CoV-2 variants tend to escape binding by many members of the groups associated with most potent neutralizers [80,81]. Notably, the epitopes targeted by RBD-5 to RBD-7 antibodies are highly conserved but less potent in the *Sarbecovirus* subgenus of *Betacoronavirus*; therefore, they can be engineered in multivalent format to achieve enhanced potency and further use in the variant-resistant cocktails formulation [80]. For example, RBD-5 to RBD-7 or Class 4 antibodies, such as S2X259 (PDB: 7RA8 and 7RAL) [103] and DH1047 [104], are broadly protective antibodies, which bind to an epitope that is highly conserved among the *Sarbecovirus* subgenus. Although nAbs are an important defense against viral infections, they are not the only mechanism of protection. The T cells responses and non-nAbs are additional measures to protect from viral infections [123,124]. Pfizer-BioNTech and Moderna mRNA vaccines for SARS-CoV-2 have been shown to elicit cross-protective B and T cell responses, which can recognize different SARS-CoV-2 variants and are minimally affected by mutations in the spike protein [125–128]. This suggests that vaccine-induced immunity will continue to protect against currently circulating SARS-CoV-2 variants. The SARS-CoV-2 virus will continue to evolve, resulting in the emergence of escape variants; therefore, worldwide genomic surveillance, better vaccination drive, development of bnAbs, and new drugs are vital to combat COVID-19.

## Supporting information

S1 Table Therapeutic countermeasures against COVID-19 under development. The data have been taken from the US Department of Health and Human Services (https://www.medicalcountermeasures.gov/app/barda/coronavirus/COVID19.aspx?filter=therapeutic).
(TIF)
