# Supplementary figures and images for: Structural and antigenic variations in the spike protein of emerging SARS-CoV-2 variants
Source: PLoS Pathog. 2022 Feb 17;18(2):e1010260. doi: 10.1371/journal.ppat.1010260 (PMC8853550; doi:10.1371/journal.ppat.1010260)

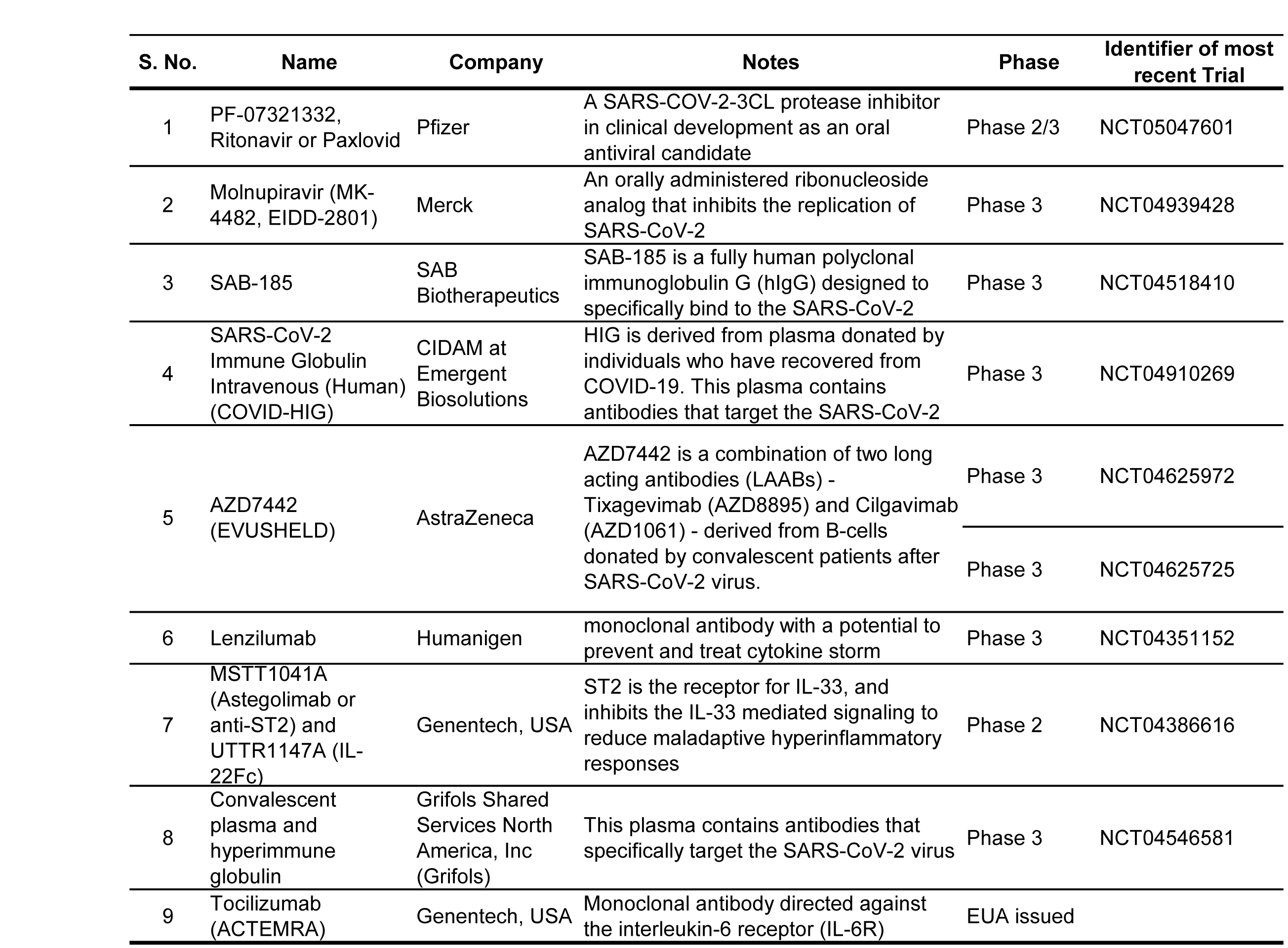

Supplement: S1 Table — The data have been taken from the US Department of Health and Human Services (https://www.medicalcountermeasures.gov/app/barda/coronavirus/COVID19.aspx?filter=therapeutic). (TIF) [file ppat.1010260.s001.tif]
